# Supplementary material for: Exercise Levels and Preferences in Cancer Patients: A Cross-Sectional Study
Source: Int J Environ Res Public Health. 2020 Jul 24;17(15):5351. doi: 10.3390/ijerph17155351 (PMC7432474; doi:10.3390/ijerph17155351)
Supplement: Supplementary file 1 [file ijerph-17-05351-s001.pdf]

SM1: Information leaflet for patients

INFORMATION LEAFLET

Study title: **Determinants of cancer patients' adherence to an exercise program**

Protocol Code: STIP ON

Principal investigator Prof. Massimo Lanza,

Co-Investigator: Dr. Sara Pilotto

Operational Unit: UOC Oncology

Dear Madam / Dear Sir,

We are writing to ask you to participate in a study promoted by the Oncology Unit that aims to identify preferences regarding various aspects of exercise. Before you make any decision, we want to be sure you understand the reason for the study and what you will be asked to do if you decide to take part. The researchers and their colleagues are available to provide any clarification you may need. This leaflet gives you the complete correct information to enable you to make your choice feely and consciously.

The researcher/professional responsible for information is Prof. Massimo Lanza, associate professor at the University of Verona.

What is the purpose of the study?

This study aims to identify the preferences of people like you, with different oncological diseases, about how and when you can take part in an adapted exercise program.

Why was I selected?

You have a malignancy, and the researchers think you have the necessary qualifications to enter the study.

Do I have to participate?

No. The decision to take part depends only on you. It is completely voluntary. If you prefer not to participate, you need not give any explanation. You will still receive all the examinations, visits, and treatments currently available for your disease.

What will happen if I decide to participate?

If you wish to consider taking part in the study, you will be given this information sheet to read and keep. You will be free to ask for any explanation you want about it. You will be asked to sign the consent form attached. Only after you have signed the consent form will you start the study and be given a questionnaire.

How will the study take place? What will I need to do?

For the study to start you will be given a questionnaire, with closed and open questions, enquiring about your preferences on how and when you exercise.

What benefits can I expect from the study?

There are no particular benefits as it only involves answering a questionnaire, but your replies will help us find the best way to encourage adapted physical activity for cancer patients.

What might be the risks, side effects, inconvenience?

There are no risks or side effects since it is only a questionnaire.

How long does the study last?

It should take you approximately 20 minutes to complete the study.

Will I have to pay additional fees?

No. Your participation will not entail any additional expense.

Who organizes and finances the study?

The study is organized by the University of Verona, in collaboration with the Azienda Ospedaliera Universitaria Integrata, University of Verona.

Are there any refunds or allowances for my participation?

There is no refund.

Who reviewed the study?

The study protocol was drawn up in accordance with the European Union's Standards of Good Clinical Practice, in accordance with the Declaration of Helsinki, and was approved by the Independent Ethics Committee of the Verona Hospital.

Will my participation remain confidential? How will my personal data be used?

Your participation will remain anonymous.

How will the results be used/disseminated?

At the end of the study, the results may be published or disseminated in an aggregate form, and you will remain anonymous.

Who can I contact for more information?

If you want any additional clarification about the questionnaire, you can contact the principal investigator Prof. Massimo Lanza, whose telephone number is XXXXXX, every day, from 10 a.m. to 4 p.m.

SM2: Questionnaire to collect cancer patient's preferences and characteristics associated with exercise

1. Date of birth: ______________________
2. Sex: □ male □ female
3. Weight: ___________ (kg)
4. Height: ___________ (cm)
5. Province of residence: _______________________
6. Education level: □ elementary (up to age 10-11 years) □ secondary (up to age 14 years) □ secondary (up to age 18-19 years) □ college/university □ postgraduate
7. Marital status: □ single □ married □ divorced □ widow/widower
8. Employment: □ retired □ homemaker □ part-time employed □ full-time employed □ other
9. Does your monthly income cover your monthly expenditure? □ inadequate □ barely adequate □ adequate □ more than adequate
10. Considering a 7-day period (a week), how many times on the average do you do the following kinds of exercise for more than 15 minutes during your free time (write in each line the appropriate number):

	Number per week
a) Strenuous exercise (heart beats rapidly) (i.e. running, jogging, hockey, football, soccer, squash, basketball, cross country, skiing, judo, roller skating, vigorous swimming, vigorous long-distance bicycling)	__________
b) Moderate exercise (not exhausting) (i.e. fast walking, baseball, tennis, easy bicycling, volleyball, badminton, easy swimming, alpine skiing, popular and folk dancing)	__________
c) Mild exercise (minimal effort) (i.e. yoga, archery, fishing from river bank, bowling, horseshoes, golf, snowmobiling, easy walking)	__________

11. Considering a 7-day period (a week) during your leisure time how often do you engage in any regular activity long enough to work up a sweat (heart beats rapidly)? □ often □ sometimes □ never/rarely

12. Are you interested in participating in an exercise program designed for cancer patients? □ yes □ no □ maybe
13. Who would you prefer to receive exercise instructions from? □ oncologist □ nurse □ kinesiologist □ nutritionist □ physiotherapist □ a cancer survivor □ no preference □ other (specify)___________________
14. How would you prefer to receive exercise instructions? □ face to face □ by telephone □ videotape □ television □ brochure/pamphlet □ radio □ over the internet □ other (specify)___________________
15. Who would you prefer to exercise with? □ nobody □ other cancer patients □ family members □ friends □ a group □ no preference □ other (specify)________________________
16. Where would you prefer to exercise? □ at home □ at a community fitness center □ at an adapted exercise fitness center □ outside □ no preference □ other (specify) __________________________
17. What time of the day would you prefer to exercise? □ morning □ afternoon □ evening □ no preference
18. What type of exercise would you prefer in summer? (list the top three)
    i. __________________
    ii. __________________
    iii. __________________
19. What type of exercise would you prefer in winter? (list the top three)
    i. __________________
    ii. __________________
    iii. __________________
20. How often would you prefer to exercise? __________________
21. In which part of the week would you prefer to exercise? □ weekday □ weekend □ no preference
22. Would you like session content to vary? □ same each time □ different each time □ no preference
23. What intensity would you prefer to exercise? □ mild □ moderate □ strenuous □ no preference
24. How would you prefer to perform exercises? □ supervised □ unsupervised □ no preference
25. What kind of exercise program would you prefer? □ individual with a program to perform at home □ individual with a personal trainer □ in a group with a kinesiologist □ none of these

26. Who would you want as "helper" during the program? □none □ exercise specialist □ neighbour □ colleague □ friend □son/daughter □ spouse □ other family member

27. Which is your cancer site? □ colon-rectum □ lung □ breast □ head-neck □ upper gastro-intestine (pancreas, esophagus, stomach, bowel, liver, biliary tract) □ gynecologic (uterus, ovary, endometrium) □ genitourinary (prostate, kidney, bladder, testicle) □ melanoma □ hematologic (lymphoma, myeloma, leukemia)

28. Which is the current status of your cancer? □ early □ advanced □ metastatic □ cured/remission □ I do not know

29. Which types of treatment do you underwent/undergoing? □ surgery □ chemotherapy □ radiotherapy □ hormonotherapy □ marrow transplant □ other (specify)___________________________________

30. When (month and year) was your cancer diagnosed? __________________/_______________________

31. The chemo/radio/hormonotherapy treatment(s) for your cancer is/are… □ about to start □ ongoing □ completed □ unknown
